# Exploring the concept of surgical transition: surgical activity in the light of economic development in Sierra Leone, Liberia, Ghana and India

**DOI:** 10.3389/fsurg.2025.1629828

**Published:** 2025-08-15

**Authors:** Juul M. Bakker, Alex J. van Duinen, Priti Patil, Priyansh Nathani, Adam Gyedu, Håvard A. Adde, Pranav Bhushan, Nobhojit Roy, Anita Gadgil, Håkon A. Bolkan

**Affiliations:** ^1^ Department of Public Health and Nursing, Norwegian University of Science and Technology (NTNU), Trondheim, Norway; ^2^ CapaCare, Trondheim, Norway; ^3^ Department of Surgery, St. Olavs Hospital HF, Trondheim University Hospital, Trondheim, Norway; ^4^ Department of Surgery, ELWA Hospital, Monrovia, Liberia; ^5^ Department of Statistics, BARC Hospital, Mumbai, India; ^6^ Department of Casualty, District General Hospital, Gadchiroli, India; ^7^ WHO Collaborating Centre for Emergency, Critical and Operative Care, The George Institute for Global Health, New Delhi, India; ^8^ Department of Surgery, School of Medicine and Dentistry, Kwame Nkrumah University of Science and Technology, Kumasi, Ghana; ^9^ Kwame Nkrumah University of Science and Technology, University Hospital, Kumasi, Ghana; ^10^ Department of Surgery, Ålesund Hospital, Ålesund, Norway; ^11^ Institute of Global Public Health, University of Manitoba, Winnipeg, MB, Canada; ^12^ Department of Global Public Health, Karolinska Institutet, Stockholm, Sweden; ^13^ Centre for Leadership in Global Health, University of Global Health Equity, Butaro, Rwanda

**Keywords:** surgical volume, global surgery, human resources for health (HRH), health system, low- and lower-middle-income countries, global south, economic development, transition

## Abstract

**Introduction:**

The surgical volume indicator measures surgical activity within a population, but it does not fully untangle the details behind the statistical indicator. As health systems evolve and countries develop economically, the types of surgeries performed, providers, and levels of healthcare facilities may provide a richer understanding of changes in surgical activity. This research studied surgical activity in four diverse settings by analyzing initial data to assess trends in patient characteristics, surgical staff, case distribution, level of care, and anesthesia practices, forming the basis for a “surgical transition” framework.

**Methods:**

We conducted a secondary analysis of surgical volume data from four studies in Sierra Leone, Liberia, Ghana, and India, to assess trends in surgical distribution. Descriptive statistics were used to compare surgical volumes by population subgroups, surgical providers, case distribution, level of care, and anesthesia.

**Results:**

Findings show that countries with higher GDP per capita had greater surgical volumes, more specialist providers, and a broader, more advanced case mix. Increases in surgical volume were most notable among older age groups, gender disparities in access diminished as systems developed. In lower-income settings, a large share of surgeries were cesarean sections or other procedures for women of reproductive age, while there were more surgeries in the older population in more advanced economies. The proportion of essential surgeries, including for example surgeries for obstetric complications, abdominal emergencies and injuries, remained stable between low- and lower-middle-income countries, decreasing only with further economic development. Specialist-performed procedures increased with economic growth, resulting in greater surgical variety and complexity.

**Discussion:**

Changes in surgical volume must be understood within the broader context of societal and economic development as well as the health system. The concept of “surgical transition” highlights how demographic and socioeconomic progress is reflected in the quantity, diversity, and complexity of surgical services. As countries advance, internal priorities, such as healthcare policies, financing, infrastructure, and service delivery mechanisms, also evolve. These factors influence surgical care delivery. Each phase of the surgical transition presents different challenges and needs. Recognizing the phase of surgical transition can help guide strategies and establish realistic interim targets for the global surgical indicators, making them more actionable tools for measuring progress and comparing systems.

## 1 Introduction

In 2015, the Lancet Commission on Global Surgery (LCoGS) called for the integration of surgical care indicators in health goals and monitoring systems (1). Six surgical indicators were defined to monitor access to and provision of safe and affordable surgical care. These indicators include access to timely essential surgery, the specialist surgical workforce density, surgical volumes, perioperative mortality rate, and protection against impoverishing and catastrophic expenditure. This information is used to guide data-informed policy decisions, and for comparison of progress in access to surgical care within and between settings. The global surgery indicators were evaluated to be simple, widely applicable, and relevant to public health (2). In 2021, the indicators were revisited, resulting in the consolidation of the two economic impact indicators into one (3). For the surgical volume indicator, the targets include a met need for surgery, defined as 5,000 surgical procedures performed annually per 100,000 population by 2030, and 80% and 100% of countries tracking surgical volume data by 2020 and 2030, respectively.

The surgical volume indicator is a useful tool to assess how much of the surgical need is met. However, by itself it only provides a singular dimension of a complex system, and needs to be supplemented with information about providers, facility level of care provision, and case mix to elaborate on surgical capacity. A more in-depth description of which surgeries are performed, by who, and where, could enhance our understanding of how surgical care evolves as systems gets strengthened. A recent systematic review by Patil et al. showed that most low- and middle-income countries (LMICs) are far from reaching the LCoGS target for surgical volume and that none of the countries reached the benchmark (4). In addition, the review demonstrated an association between an increase in the GDP of a country and the surgical volume. Cesarean section (CS) and hernia, among the essential surgeries, have been considered as index surgeries to represent the volumes of surgeries performed in the countries (5–7). It was observed by Patil et al. that the proportion of CSs and hernia repairs decreased with an increase in a country's GDP, whereas the proportion of laparotomies increased with an increase in GDP (4).

This study aims to describe patterns in surgical systems across four countries at different stages of economic development. We propose the surgical transition framework to describe this strengthening of surgical systems as countries develop economically. This transition reflects a shift from limited surgical services to more accessible, comprehensive, and advanced surgical care.

## 2 Methods

This study is a secondary, descriptive analysis of pooled surgical volume data from four low- and middle-income countries (LMICs). We selected studies from Sierra Leone (8), Liberia (9), Ghana (10), and India (11), countries at different levels of economic development and where the surgical volume was recently assessed, and where data was accessible. Primary data from these studies were pooled into a database and analyzed to assess trends in the distribution of surgical procedures. Information from the selected studies, such as surgical volume and surgical specialist density, as well as economic development indicators, are presented in Table 1.

**Table 1:** Background information of the study settings.

Setting	Lindheim-Minde et al. (8)	Adde et al. (9)	Gyedu et al. (10)	Bhandarkar et al. (11)
Study information				
Study period	2017	2017–2018	2014–2015	Jan 2017–Dec 2018
Number of surgical procedures included	6,982	6,428	103,505	9,284
Study population size	7.7 mln.	4.9 mln.	28.9 mln.	88,273
Sampling method	3-month sample	4-month sample	Facility sample	All in UHC
Surgical volume (per 100 000 population)	372	462	869	5,259
Surgical specialists (per 100 000 population)	1.2	1.6	N/A	6.5^b^
Economic parameters (in study year)^a^				
Income classification	LIC	LIC	LMIC	UMIC
GDP per capita (current US$)	485	700	1,711	2,033^d^
Total health expenditure per capita (current US$)	46	69	78	79^d^
OOP expenditure (% of CHE)	54.5%	45.6%	35.8%	53.2%^c^
Demographic and health parameters^a^				
Population size (in million inhabitants)	8.8	5.4	34.1	12.7^d^
Life expectancy (years)	59	61	63	71^c^
Birth rate (per 1,000 population)	34	33	32	12^d^
Death rate (per 1,000 population)	10	9	8	7^d^
MMR (per 100,000 live births)	520	684	286	88^d^

CHE, current health expenditure; GDP, gross domestic product; mln., million; OOP, out-of-pocket; PPP, purchasing power parity.

^a^ Source except specified otherwise: World Bank Open Data (12).

^b^ Based on Holmer et al. (59).

^c^ For India as a whole, not Mumbai-specific data.

^d^ Based on data from Mumbai Corporation of Greater Mumbai (60), Reserve Bank of India, and National Health Accounts 2017–18.

### 2.1 Study setting and data collection methods

#### 2.1.1 Sierra Leone

Sierra Leone is a low-income country in West-Africa with 7.7 million inhabitants in 2017 (12). A study by Lindheim-Minde et al. examined the nationwide surgical activity in Sierra Leone in 2017 and documented a surgical volume of 372 per 100,000 population in 2017, whereas this was 400 in 2012 (8, 13). In 2017, surgical volume data were collected from operating theaters and delivery registration logbooks from 49 out of 60 public and private facilities nationwide that were identified to perform surgeries. The included facilities were 11 clinics/health centers, 29 first-level hospitals, and 9 referral or specialty hospitals. Detailed surgical procedure data were collected for three months (February, June, October) and extrapolated to annual numbers. Population projections for 2017 were derived from the national census in 2015 (14). Surgical volume data was categorized into specialty categories, such as general surgery, obstetrics and gynecology, orthopedics, and ophthalmology.

#### 2.1.2 Liberia

Liberia is a low-income country in West-Africa, and a neighboring country to Sierra Leone, with 4.9 million inhabitants at the time of the study (12). The selected study by Adde et al. enumerated surgical volumes and quantified the availability of surgical infrastructure, personnel, and essential surgical procedure in Liberia using similar methodology as in Sierra Leone. The surgical volume in 2017–2018 was 462 per 100,000 population (9). A four-month sample between October 2017 and July 2018 from 51 out of 52 healthcare facilities nationwide that were used to calculate annual surgical operations. The health facilities included both public and private clinics and hospitals and consisted of 16 clinics/health centers, 30 first-level hospitals, and 5 referral hospitals. Data on surgical procedures were retrieved from operating theater surgical and anesthesia logbooks.

#### 2.1.3 Ghana

Ghana is a lower-middle income country in West-Africa with a population size of 28.9 million in 2015 (12). The selected study by Gyedu et al. aimed to evaluate and characterize the operation rate in Ghana. It involved a retrospective review of operation logbook data between June 2014 and May 2015 from a representative sample of district hospitals (48 of 124), regional hospitals (9 of 11) and tertiary hospitals (3 of 5) (10). The national surgical volume was estimated by applying a probability weight for each of the hospital-level strata. The surgical volume was 869 per 100,000 population. Surgical procedures were further categorized by hospital level and by surgical procedure type using the DCP3 classification (7).

#### 2.1.4 India (Mumbai)

The study by Bhandarkar et al. examined an urban cohort of people covered by an employees’ health scheme in Mumbai to identify the surgical volume and analyze surgical needs (11). Mumbai is the capital of Maharashtra, a state of India. The Mumbai Metropolitan Region had an estimated population of 20 million in 2018 (15). Even though India is a lower-middle income country, Mumbai can be considered to be upper-middle income level (16). The study analyzed the surgical volume between January 2017 and December 2018, in a cohort of the population (88,273 people) covered by a ‘Contributory Health Service Scheme’ (CHSS) for employees. Surgical procedure data were collected from the centralized Electronic Medical Records used in the facilities and the cashless vouchers for care availed by members for specialty treatment outside the CHSS system. Surgical procedures were performed in the health scheme's central hospital, with certain subspecialist procedures being outsourced to a tertiary hospital. This data was used to calculate the surgical needs in the population after standardizing for age and sex according to the national census data from 2011. Surgeries were categorized by specialties and as “essential” or “non-essential” based on the DCP3 classification. The surgical volume in this cohort was 4,642, resulting in a national estimated volume of 5,259 per 100,000 population.

### 2.2 Definitions and data processing

In this study, we pooled surgical procedure data from the four index studies described. Data were extrapolated to present the annual surgical volume. As described under “study setting”, no uniform definition of “surgical procedure” was used across the settings. For all, data included emergency as well as planned procedures, but not bedside and emergency room procedures.

Surgical provider definitions were as follows: for specialists, we included any person who has completed postgraduate training in a surgical specialization, but not residents in training, who were categorized as non-specialist medical doctors. Associate clinicians included any other trained health professional, such as surgically trained community health officers in Sierra Leone (SACHOs), physician assistants, obstetric clinicians (Liberia), midwives, and nurses.

To account for heterogeneity in the data, all surgical procedures in the combined database were re-categorized using the DCP3 classification (7). The DCP3 essential surgical procedures are divided between primary, secondary, and tertiary levels of care. All procedures were categorized to the DCP3 classification by two authors independently (JB, HB). In the same manner, all procedures were categorized into a classification based on surgical subspecialties (PN, AvD, PB, AG). Conflicts were resolved by a third person (NR, HB) and, if not conclusive, discussed in the research team.

The level of care ranged from health center/clinic, first-level hospital (district), and referral and specialty hospitals (regional, tertiary, and specialty hospitals). Health centers and clinics were only included in the datasets from Sierra Leone and Liberia. For Ghana, numbers were adjusted for the included sample hospitals at each level.

### 2.3 Data analysis

We analyzed the data using descriptive statistics for differences and trends in surgical volume by population subgroups (age, sex), surgical provider contributions, the case distribution and the level of care. Data analysis was performed in Microsoft Excel.

For each setting, population data disaggregated by age and sex was obtained for the study year and the surgical volumes were stratified by age and sex (15, 17). The surgical volume rate for each group was calculated as:

$$\mathrm{Age}/\mathrm{Sex}\;\mathrm{specific}\;\mathrm{Surgical}\;\mathrm{Volume}\;\mathrm{Rate}\;=\frac{\;\mathrm{Surgical}\;\mathrm{Procedures}\;\mathrm{in}\;\mathrm{Age}/\mathrm{Sex}\;\mathrm{Group}\;}{\mathrm{Population}\;\mathrm{Size}\;\mathrm{of}\;\mathrm{Same}\;\mathrm{Group}}\times \;100,000$$

This normalization to a rate per 100,000 population and to a one-year period enabled comparison of age/sex patterns across countries. To visualize these patterns, a population pyramid showing the demographic distribution by age and sex was combined with a scatter plot to display the relationship between the population size and the corresponding surgical volume rate in each age/sex group.

The proportion of surgical procedures performed by different surgical cadres, such as specialists, non-specialist medical doctors, and associate clinicians, was calculated and compared for each setting. The same was done for facility levels of care. In addition, we compared the anesthesia techniques used during surgical procedures in the four countries, differentiating between local anesthesia, regional anesthesia, ketamine, and general anesthesia.

## 3 Results

### 3.1 Surgical volume by demographic groups

**Figure 1:**
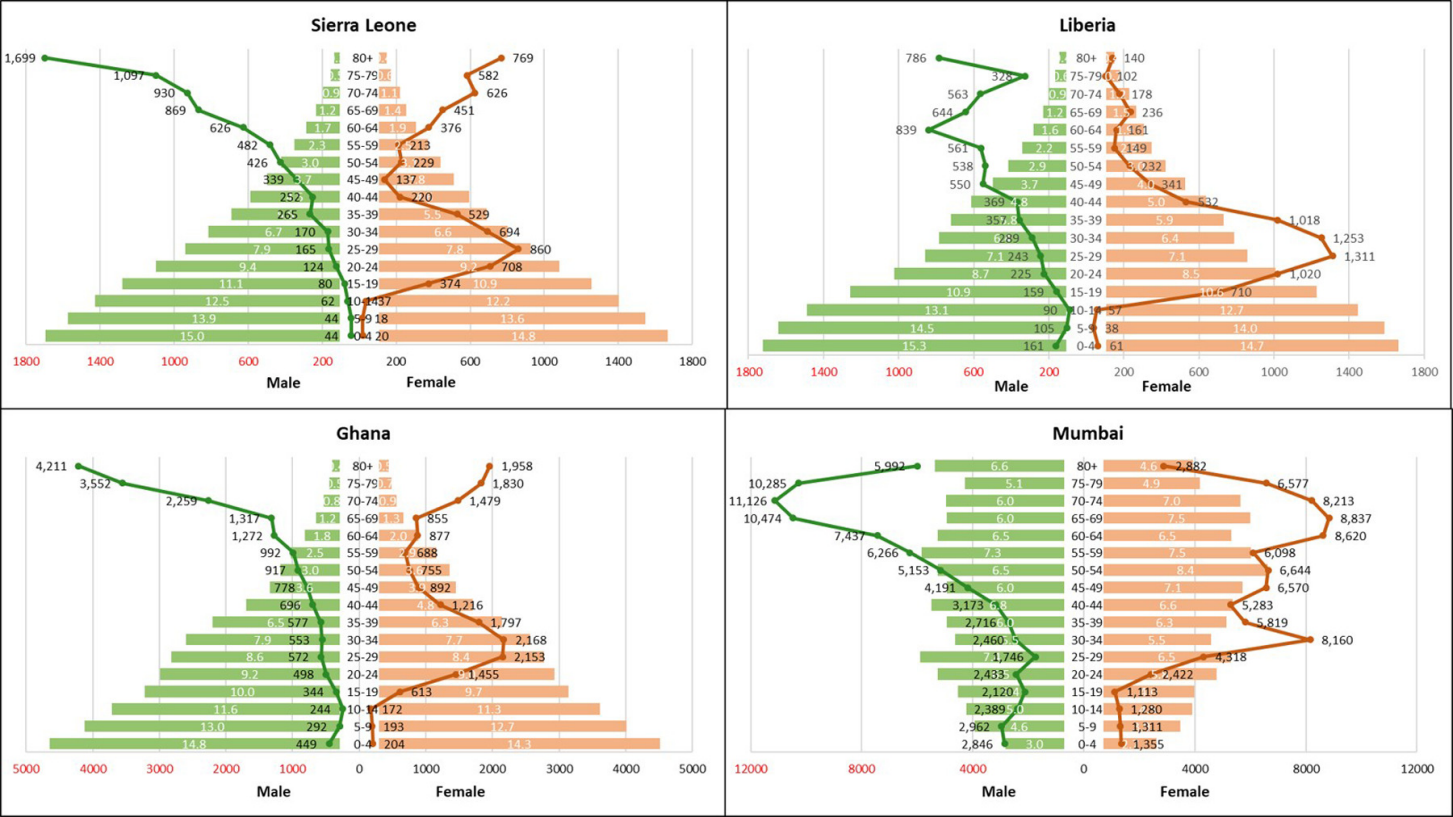
Populationpyramids and surgical volume distribution for Liberia, Sierra Leone, Ghana and mumbai. Surgical volume per 100,000 population. Bars = population pyramid, % by gender and age group. Lines = surgical volume by gender and age group.

Surgical volume varied substantially across different age/sex-groups. Surgical volume was higher within older age groups, with the highest surgical volume in the age groups >50 years (Figure 1). For women, an additional increase in surgical volume was seen in reproductive age groups with a peak around 25–35 years.

In Sierra Leone, Liberia and Ghana the surgical volume decreased after the reproductive age, whereas in Mumbai the volume remained between 5,000–9,000 operations per 100,000 population up to 80 years of age. In Sierra Leone and Ghana, the surgical volume for women was the highest in the reproductive age groups as well as at an older age, whereas in Liberia the highest surgical volume for women was only observed in the fertile age groups. However, in all settings the surgical volume was higher for men than for women in the age groups above 60. In this age group, the three most common procedures were cataract operations, hernia repair, and catheterization, of which the latter two were more prevalent in men than in women.

### 3.2 Surgical volume by surgical provider cadre

**Figure 2:**
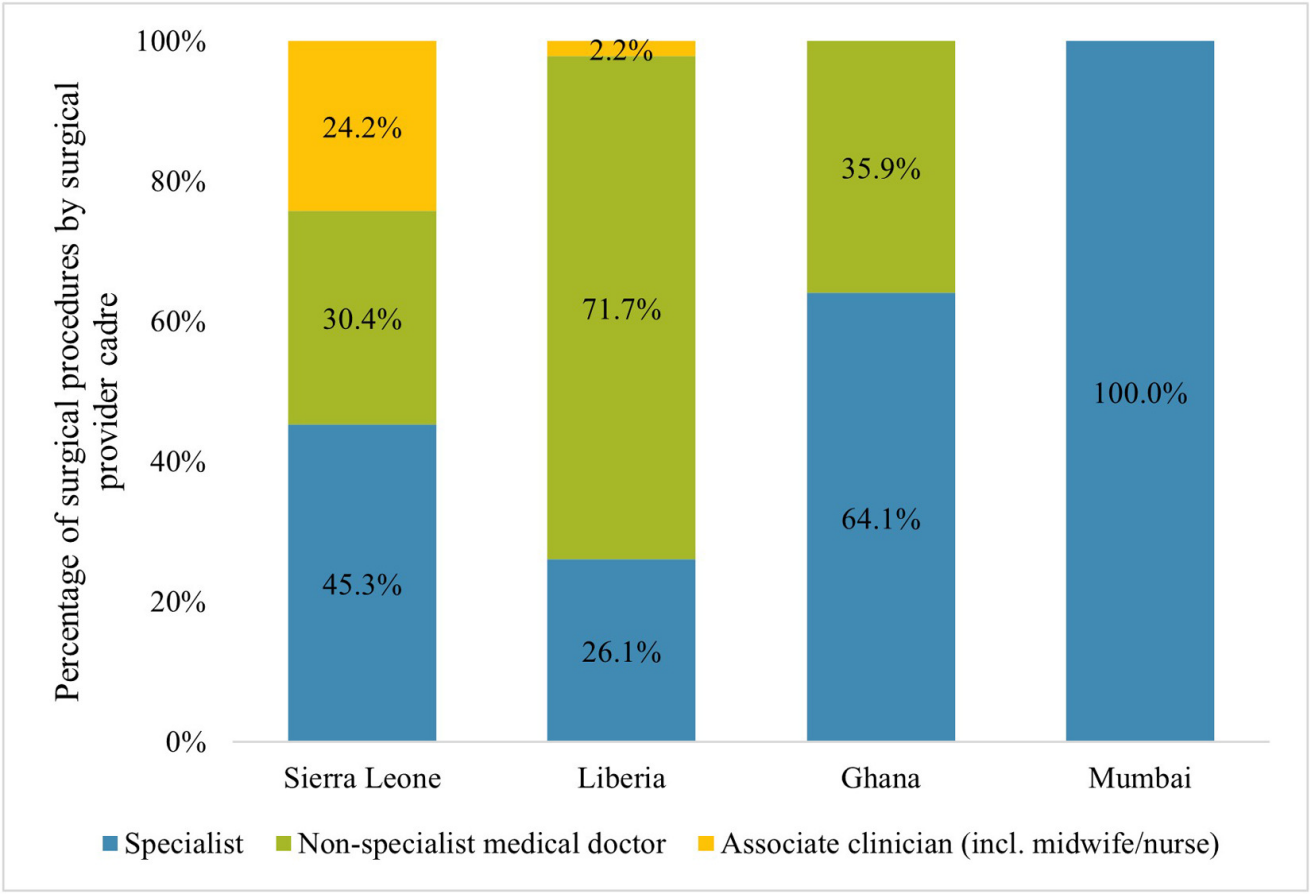
Distribution of surgeries by type of provider*. *Assuming similar division of providers for procedures where the provider was unknown.

Countries with a higher GDP had a larger proportion of surgical procedures performed by specialists (Figure 2). In Liberia and Sierra Leone, most surgical procedures were performed by non-specialists, whereas most procedures in Ghana and all in Mumbai were performed by specialists. In Sierra Leone, almost a quarter of procedures (24.2%) were performed by surgically trained associate clinicians. The percentage of surgical procedures performed by associate clinicians was only 2.2% in Liberia. Despite a large proportion (46.2%) of missing surgical provider data from Ghana, the available data indicate that no surgical procedures were performed by associate clinicians.

### 3.3 Case distribution

**Figure 3:**
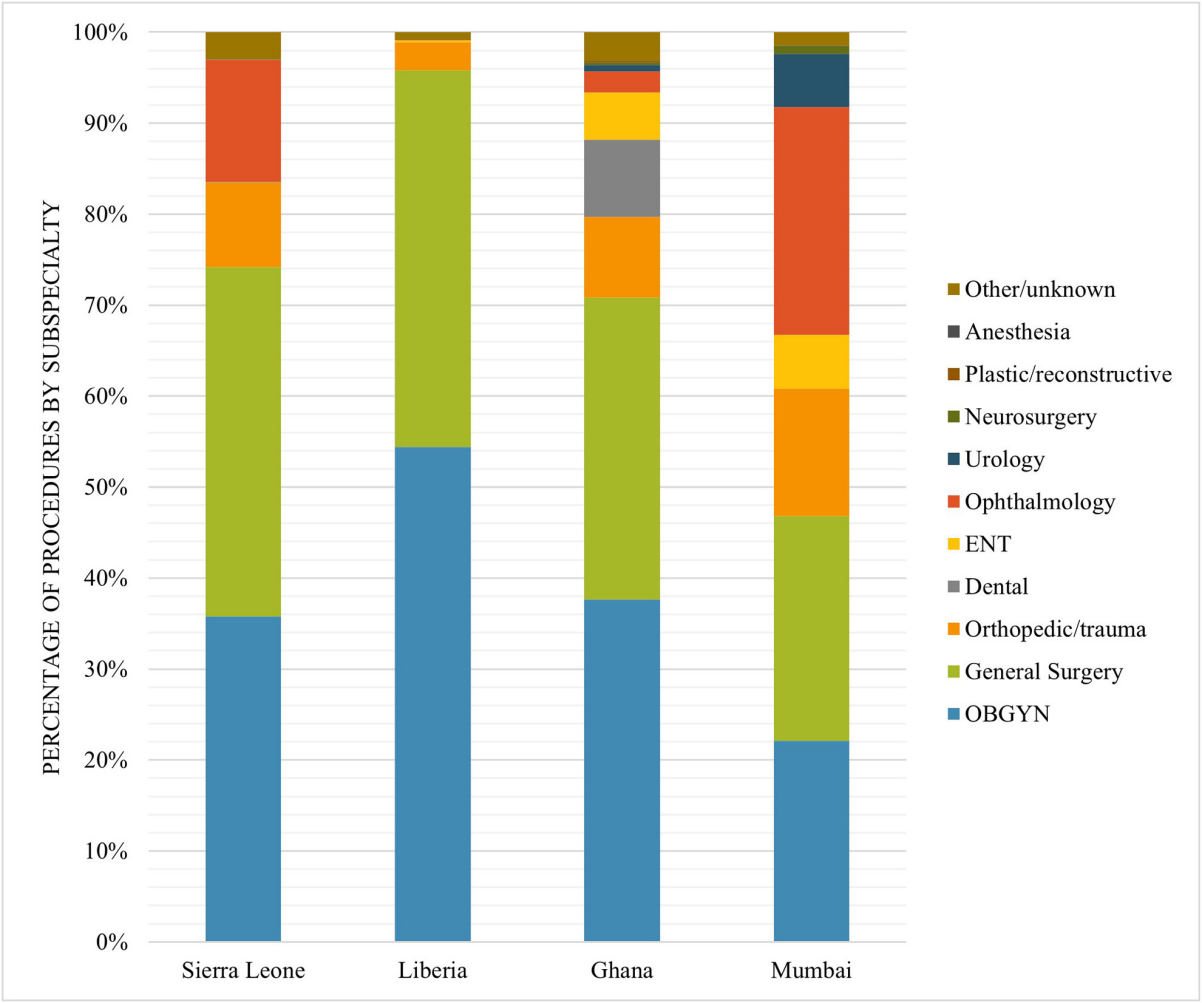
Case distribution of surgical procedures by subspecialty by setting.

The most frequently performed surgical procedures across all settings were in general surgery and in obstetrics & gynecology (Figure 3). A more diverse case distribution was observed in settings with higher GDP per capita. In Ghana, Liberia and Sierra Leone, most surgical procedures were essential procedures according to the DCP3 classification (79.3, 89.7, and 71.1% respectively), whereas in Mumbai this was 47.7%. A detailed overview of all surgical procedures according to the DCP3 classification is presented in Supplementary Table S1. CS and hernia surgeries were among the 10 most performed surgical procedures in each of the settings (Table 2). The CS to total operation ratio (CSR) was 0.28 in Sierra Leone, 0.43 in Liberia, 0.26 in Ghana, and 0.04 in Mumbai. Suturing laceration and cataract surgery were among the 10 most performed procedures in Sierra Leone, Liberia and Ghana. Fracture reduction and manual vacuum aspiration/dilatation and curettage were in the 10 most performed procedures in Mumbai and Ghana, but not in Liberia and Sierra Leone.

**Table 2:** Top 10 procedures in Sierra Leone, Liberia, Ghana, and Mumbai.

Sierra Leone	%	Liberia	%
1. Cesarean section	27.6%	1. Cesarean section	43.1%
2. Hernia repair	16.0%	2. Hernia repair	21.2%
3. General surgery—other	9.5%	3. Obstetrics & Gynecology—other	4.2%
4. Orthopedic surgery -other	8.5%	4. Laparotomy	4.2%
5. Cataract extraction and insertion of intraocular lens	8.3%	5. General surgery—other	4.0%
6. Ophthalmic surgery other	5.1%	6. Dilatation and curettage	2.8%
7. Appendectomy	3.7%	7. Abscess incision	2.6%
8. Unknown	2.9%	8. Appendectomy	2.3%
9. Obstetrics & Gynecology—other	2.5%	9. Ectopic pregnancy	2.1%
10. Laparotomy	2.4%	10. Hysterectomy	2.0%
Top-10 procedures	86.4%	Top-10 procedures	88.5%
Ghana	%	Mumbai	%
1. Cesarean section	26.0%	1. Cataract extraction and insertion of intraocular lens	23.3%
2. Dental procedures	8.5%	2. Obstetrics & Gynecology—other	13.2%
3. Suturing laceration	7.3%	3. Orthopedic surgery -other	10.4%
4. Hernia repair	7.2%	4. Endoscopy	8.7%
5. ENT procedures	5.2%	5. ENT procedures	5.9%
6. Obstetrics & Gynecology—other	4.5%	6. Urology procedures	5.8%
7. Dilatation and curettage	4.3%	7. Cesarean section	3.9%
8. Fracture treatment—conservative	4.3%	8. Fracture treatment—operative	3.2%
9. Abscess incision	3.8%	9. Hernia repair	2.9%
10. Fracture treatment—operative	3.3%	10. Oncology (general surgery)	2.5%
Top-10 procedures	74.4%	Top-10 procedures	79.9%

### 3.4 Distribution of surgical volume by level of care

In Sierra Leone and Liberia data included all levels of care where surgery was performed, ranging from health center level to tertiary level, whereas in Ghana and Mumbai only hospitals were included. Most surgical procedures were performed in first-level hospitals, with the percentage ranging from 44.9% in Sierra Leone to 53.8% in Ghana, 61.1% in Liberia, and 89.6% in Mumbai. In both Sierra Leone and Liberia, 7.8 and 8.1% of all surgical procedures were performed in health centers or clinics (public and private). In Sierra Leone 22.7% of all procedures were performed in specialty hospitals. The division between levels of care was comparable between essential surgeries and other surgeries (Supplementary Figure S1), with only a slightly higher proportion of non-essential surgical procedures in referral and specialty hospitals in Sierra Leone and Mumbai.

### 3.5 Type of anesthesia

For Mumbai, no information about the type of anesthesia was available. Amongst the other three countries, the use of general anesthesia was comparable, with 32.2% in Sierra Leone, 27.2% in Liberia and 27.4% in Ghana. The use of Ketamine as an anesthetic drug was not specified for Ghana, this was registered in 13.7% of operations in Sierra Leone, and 3.0% in Liberia. The proportion of regional anesthesia was highest in Ghana (71.9%) and lowest in Sierra Leone (42.0%). Local anesthesia was only recorded in Liberia and Sierra Leone (8.0% and 9.8% respectively). No information was reported about anesthesia providers.

## 4 Discussion

### 4.1 Key findings

This study supports the theory that economically developed settings with a higher GDP per capita have more developed surgical systems, with higher surgical volumes, more specialist providers, and a more diverse and advanced case distribution (4, 18). In addition, we observed that the increase in surgical volume is more prominent in older age groups and that the gender difference in surgical volume reduced in all age groups. In less developed settings, a large proportion of the surgeries are CS or other obstetric and gynecological surgeries for women in the reproductive age. The proportion of essential surgical procedures remained relatively stable between the low-income (Sierra Leone, Liberia) and lower-middle income (Ghana) settings and only decreased with further economic advancement (Mumbai).

#### 4.1.1 Country-specific policies

In Sierra Leone and Liberia, we see a fragmented surgical system delivering basic health services. This includes a low surgical volume, a young population, a high fraction of essential and reproductive services, generalist-led care with few specialists, and substantial geographical and socioeconomic inequity in access to care (17). In both countries, the health system was severely affected by war in the 1990's, and the West-African Ebola Virus Disease outbreak in 2014–2016. To rebuild the health workforce, in Sierra Leone a surgical task-sharing program was started in 2011 to train community health officers in essential surgical and obstetric procedures (19). During the same time the Free Healthcare Initiative was established providing free-of-charge treatment for children under five and obstetric care (20). In addition, a national ambulance system was set up to improve access to emergency obstetric care (21). A qualitative study identifying barriers to increase surgical productivity in Sierra Leone found that the main barriers involve both patient and facility financial constraints, lack of equipment and supplies, weak regulation of providers and facilities and a small surgical workforce, which experiences a lack of recognition (22).

In Liberia, the main aims of the Government post-civil war and -Ebola was to improve access to safe and quality health services, especially for the vulnerable populations, through the delivery of a package of essential health services (23). Investments were made to upgrade health facilities, to improve health facility infrastructure, and to build a resilient and responsive health workforce. This included establishment of a postgraduate training program to train a specialist workforce (24), increasing capacity at the only medical school in the country to increase the number of general practitioners, as well as establishing programs to train allied health workers, such as nurse anesthetists and obstetric clinicians (25–28).

In both Sierra Leone and Liberia, no National Surgical, Obstetric, and Anesthesia Plan (NSOAP) has been finalized. Although there is no financing specifically allocated to surgical care, funding is linked to broader sustainable financing mechanisms for health service delivery. Through increasing public health expenditure, emphasizing essential service packages, strengthening health infrastructure, and improving the availability of essential surgical equipment and supplies. Current health policies aim to improve the availability and quality of surgical care (29–31).

In Ghana, we see that surgical services are slightly more expanded beyond basic health services. However, most of the surgical operations performed are essential procedures and take place at primary hospitals. At district-level, there are infrastructure challenges with inconsistent access to diagnostic and treatment modalities, including surgical equipment and supplies. The Ghanaian system faces workforce inadequacies with most specialists concentrated in urban areas (32). Over the past decade, the Ministry of Health has undertaken several initiatives to strengthen surgical care across the country. In 2024, a NSOAP was launched for 2025–2029 to ensure equitable access to safe and high-quality surgical, obstetric, trauma and anesthesia (SOTA) care. The policy focuses on enhancing infrastructure, expansion and equitable distribution of the surgical workforce, improving service delivery, securing financing, and strengthening information management and governance. The plan is projected to cost approximately $503 million over its duration (33). A National Health Insurance Scheme (NHIS) that covers 68% of the Ghanaian population significantly reduced the risk of catastrophic expenditure for surgical care. However, up to 91% of the direct costs are paid out-of-pocket (32).

Lastly, in Mumbai, advanced and comprehensive health services are available. The surgical volume is a lot higher, and surgery is performed over a broader range of (sub)specialisms. However, the data in this study represents a specific patient population, who are covered by an insurance scheme. On a national scale there is a heterogeneous distribution of surgical specialists resulting in varying surgical volumes across different regions and levels of care (34, 35). The findings here are therefore more in line with what is expected in a setting with “universal health coverage”, or a high-income setting. Other tax-funded national health insurance schemes, such as Ayushman Bharat Pradhan Mantri Jan Arogya Yojana (PM-JAY) have been implemented since 2018 to provide hospitalization coverage including surgical care, for nearly 40% of the population, with a focus on economically vulnerable households (35, 36). The study included in our analysis demonstrates that coverage schemes like these are effective not only in increasing the volume of procedures, but also in expanding their variety and complexity. To achieve universal health coverage, surgical care is an essential component.

### 4.2 Surgical transition

Evolution of a surgical system, or “surgical transition”, reflects socioeconomic development. Surgical transition can provide a framework to guide surgical system development and volume research in the future. As they stand, the benchmarks for surgical volume and workforce are unrealistic for most LMICs. The concept of surgical transition could help set more nuanced interim targets as countries develop and data-driven strategies to improve their surgical care system.

Based on the three dimensions of Universal Health Coverage (population coverage, service coverage, and financial protection) and existing frameworks, such as the LCoGS surgical indicators and the Sustainable Development Goals, a surgical transition framework can be constructed (Table 3) (1, 37, 38). Surgical transition can be described in five stages, describing the development of a surgical system over time. The following elements can be identified as components of the surgical transition framework: an increase in surgical volume, an increase in the number of surgical providers with a shift from generalists to specialists, evolution from emergency surgeries to elective surgeries and from focus on basic life-saving surgeries to a wider spectrum, an improved quality, and more financial protection to access surgical care.

**Table 3:** Suggested indicators for a surgical transition framework.

Dimension of UHC	Transition phase	I	II	III	IV	V
Economic development	LIC	LMIC	LMIC-UMIC	UMIC-HIC	HIC
Population coverage	Surgical volume (1, 44)	<500	500–2,500	2,500–5,000	5,000–10,000	>10,000
	Maternal mortality rate (61, 62)	>1,000	300–000	50–299	<50	<5 (all avoidable deaths avoided)
	CS to total surgeries volume ratio (CSR) (44)	>20%	10%–20%	5%–10%	2.5%–5%	<2.5%
Service coverage	Case-mix (39, 40)	Emergency operations, limited mix of procedures	Predominantly emergency operations, limited mix of procedures	Shift to elective procedures and larger variety. Increase in non-communicable disease and injuries	Predominantly elective operations, subspecialization, more complex procedures	Increased disease chronicity and ageing
	Specialist surgical workforce per 100,000 population (1, 63)	≤2	2–9	10–19	20–40	>40
	Peri-operative mortality rate (POMR) (64)	>10%	5%–10%	1.5%–5%	0.5%–1.5%	<0.5%
Financial protection	Patient costs (58, 65)	Out-of-pocket payments, unaffordable for many	Predominantly out-of-pocket, some health insurance (government, formal sector workers)	Expansion of health insurance coverage, private sector initiatives emerging	Majority covered by national health insurance schemes	Universal health coverage. Rising costs.

### 4.3 External factors

Demographic and epidemiological changes, as well as economic development, influence the surgical needs. In this study, the increase in surgical volume in older age groups reflects the demographic transition, highlighting the transformation in the population structure of countries when they develop from high birth and death rates to low birth rates, low death rates and an increased life expectancy (39). Similarly, the epidemiologic transition describes the changing patterns of health and disease when societies develop (40). The socioeconomic, demographic and epidemiologic development of countries will influence the needs for surgery, as well as the capacity to provide surgical care (41–43). In Ghana and Mumbai, the birth and death rates are lower than in Sierra Leone and Liberia, leading to an increase in the proportion of the aging population. This development transforms the disease burden, with an increase in chronic and non-communicable diseases and a decrease in infectious diseases, thereby also changing the surgical demand. As we observed, in older age groups procedures for cataract, hernia, and prostate problems were most common.

The relatively high surgical volume among women of reproductive age mirrors both the demography and epidemiology of low-income countries, with generally higher fertility rates, but also how those surgeries are prioritized in lower resourced countries. Although overall numbers are low, limited availability and use of antenatal care may result in proportionally high numbers of unplanned CSs. It has been established that the CS to total surgeries ratio (CSR) is generally higher in low-income countries than in high-income countries and with lower health expenditure (6, 44). Weiser et al. described a decrease in the proportion of CS from 29.6% in countries with very low health expenditure to 2.7% in the high expenditure group (44). A CSR of more than 0.2 is associated with insufficient surgical capacity, acknowledging that in resource-constraints settings emergency procedures are prioritized over elective ones. This relates to the observation in this paper, where only Mumbai had a CSR <0.2.

### 4.4 Structure of surgical care

A country's healthcare infrastructure, policies, and service delivery mechanisms transform with socioeconomic progress. This healthcare system transition involves changes in how healthcare is financed, organized, and provided, ultimately aiming to improve access, quality, and equity of healthcare services (45). Key factors behind the current failure of health systems to provide adequate surgical care in most LMICs include lack of prioritization, insufficient investments and weak governance (46).

More economically developed settings deliver more varied and complex surgical care. Surgical procedures such as laparoscopy and endoscopy require a more complex surgical system, including better infrastructure and a more specialized health workforce. At the same time, the demands from the surgical system will change. When larger proportions of the population can afford surgical care, there will be a shift to more elective and advanced surgeries being offered. Within the surgical transition concept, the roles of generalist and specialist surgical providers are crucial and must be carefully balanced to meet the evolving needs of the health system. We observed a clear increase in the proportion of surgeries by specialists from low- to higher-income settings. This shift from generalist to specialist providers may reflect changes in demand, capacity, and health priorities. In low-resource settings, such as Sierra Leone and Liberia, generalist providers, such as associate clinicians or general practitioners trained in essential surgical procedures, are critical in addressing the high unmet need for basic surgical care (47). The challenges LICs face when advancing their surgical transition towards more specialist care are cost and equity. Training of generalist providers is often more cost-effective (1), and generalists are more likely to work in rural and underserved areas, bridging geographic disparities in surgical care (48). Medical and postgraduate education should therefore be aligned with, or ideally ahead of, the development of the surgical system. In addition, the backlog of unmet surgical procedures over the past decades will influence the volume and type of surgeries needed (49). In areas where there is a lack of medical doctors, certain procedures could be safely performed by associate clinicians as seen in Sierra Leone (50, 51). Challenges to generalist-driven systems are quality assurance, which requires robust training, supervision and regulation (47). As in the case of Mumbai, in high-resource settings, surgical systems prioritize specialization. Generalists continue to play a supportive role in primary and emergency care while specialists bring expertise and advanced skills to manage a greater variety and complexity of surgical conditions, improve surgical outcomes, and drive advancements in surgical techniques, technologies and overall standard of care (52, 53). Hereby it should be recognised that the various indicators of the surgical transition framework are interconnected. For instance, the lower peri-operative mortality rate in high-income countries compared to low-income countries is not completely attributable to greater access to specialist care, but also to differences in case distribution as exemplified by the higher proportion of obstetric procedures in low-income countries.

For all four settings, we observed that the first-level hospitals (district hospitals) are the backbone of the surgical system. As a first, and often only, level of access for patients in need of surgical care, first-level hospitals have an essential role to achieve universal health coverage. For simple essential surgeries such as hernia repair, first-level hospitals are as safe and effective as larger secondary and tertiary hospitals (54). Despite this important role in the delivery of essential services, they are often not well equipped in terms of personnel and resources to utilize their full potential (55, 56). Adequate investment in resources and workforce in first-level hospitals will improve equitable service coverage, while subsequently reducing the burden on referral hospitals.

Underprioritization of surgical care does not only happen at policy level; also many patients in LMICs do not prioritize seeking treatment for chronic or non-life-threatening conditions, such as non-communicable diseases and cancer. Out-of-pocket costs are an important obstacle for seeking surgical care. Globally, 43.9% of the population is at risk of incurring catastrophic expenditure due to direct costs of surgery (57). Furthermore, even in settings with policies for free healthcare, patients often face substantial indirect costs, such as transportation, food, accommodation for accompanying family members, loss of income, and informal payments (58). To increase financing for surgical services, greater commitments from governments to mobilize resources mobilization and adequate budgetary allocations, as well as pooling mechanisms and risk protection are essential (46, 58).

### 4.5 Strengths and limitations

By analyzing surgical care systems in four different Global South settings, this study highlights the distinct needs of each context. The proposed surgical transition framework introduces additional indicators and perspectives to understand the evolution of surgical care systems. Together, these insights can assist countries in developing strategies to strengthen their surgical care systems and surgical training programs. A notable strength of this study is the use of primary datasets, which enhances data quality. The inclusion of patient, provider and procedure information across all four datasets provided a unique opportunity to generate insights into the process of surgical development and transition. However, this study also has several limitations. The GDP-based comparison does not account for wealth distribution within countries and may therefore mask inequities. There were differences between the settings in terms of data collection methods, periods and duration, as well as facility selection. Each methodology had its own limitations and may not have adequately represented the volume and variations in surgical cases, e.g., by seasonal variations or epidemiological patterns influencing health facility workloads. These factors could potentially have impacted the study outcomes. The use of different procedure and facility classification systems in the primary data across settings compromised comparison. In Ghana, the private sector was excluded as it was not known to contribute significantly to national surgical volume (10). The Liberia database did not contain ophthalmology procedures. Missing data could not be retrieved, and the Ghana and Mumbai databases lacked original theatre book data but were already (re)classified in the electronic databases, restricting granularity of the analysis. The Mumbai data was specific to an urban cohort, limiting generalizability. In addition, surgical volumes as presented here only reflect patients who accessed care, omitting those unable to overcome barriers in seeking, reaching and receiving care. Finally, the study included data from just four settings. Data collection occurred over various time periods, with some data gathered a decade ago, not reflecting recent developments in the surgical system. More diverse and recent datasets from different geographic settings are needed to validate and further develop the concept of surgical transition.

### 4.6 Recommendations

To progress towards universal access to safe, affordable surgical and anesthesia care when needed, several governments have started developing and implementing NSOAPs. The availability of registry data and mappings, as utilized in this study, are essential for effective planning. The concept of “surgical transition” is relevant for context-adapted, data-driven decision-making, as each transition phase presents distinct challenges and needs. Recognizing the current phase of a country's surgical transition, as well as understanding how this process unfolds, enables clearer identification of the next steps in the development process. Since the targets set by the LCoGS are currently too ambitious for many low-income countries, the surgical transition model proposed here provides a stepwise approach, facilitating the establishment of context-specific and achievable targets aligned with the development level of a country.

## 5 Conclusion

While surgical system improvement can be measured by surgical volume, additional indicators, such as the cases performed and prioritized, the availability of specialists to perform surgeries, and the level of facilities that can undertake these surgeries can add aspects to measure and track improvements in surgical systems. The surgical transition framework describes these changes and relates them to demographic and economic changes and context-specific policies. The shift from low surgical volumes and generalist-led surgical care in low-income countries to an older population with more chronic, non-communicable conditions when countries advance economically, necessitates a system capable of handling a wider variety and complexity of surgical operations. The concept of surgical transition, by recognizing countries’ varying needs, can guide the development of achievable roadmaps to improve surgical systems.

## Data Availability

Datasets can be made available upon reasonable request. Requests to access these datasets should be directed to juul.m.bakker@ntnu.no.
