# Supplementary material for: Exploring the concept of surgical transition: surgical activity in the light of economic development in Sierra Leone, Liberia, Ghana and India
Source: Front Surg. 2025 Aug 15;12:1629828. doi: 10.3389/fsurg.2025.1629828 (PMC12394221; doi:10.3389/fsurg.2025.1629828)
Supplement: Supplementary file 1 [file Datasheet1.pdf]

CARE case report guidelines

CARE Checklist of information to include when writing a case report

(cc) BY-NC-ND

Topic	Item	Checklist item description	Reported on Line
**Title**	**1**	The diagnosis or intervention of primary focus followed by the words "case report"	√
**Key Words**	**2**	2 to 5 key words that identify diagnoses or interventions in this case report, including "case report"	√
**Abstract (no references)**	**3a**	Introduction: What is unique about this case and what does it add to the scientific literature?	√
	**3b**	Main symptoms and/or important clinical findings	√
	**3c**	The main diagnoses, therapeutic interventions, and outcomes	√
	**3d**	Conclusion—What is the main "take-away" lesson(s) from this case?	√
**Introduction**	**4**	One or two paragraphs summarizing why this case is unique (**may include references**)	√
**Patient Information**	**5a**	De-identified patient specific information.	√
	**5b**	Primary concerns and symptoms of the patient.	√
	**5c**	Medical, family, and psycho-social history including relevant genetic information	√
	**5d**	Relevant past interventions with outcomes	√
**Clinical Findings**	**6**	Describe significant physical examination (PE) and important clinical findings.	√
**Timeline**	**7**	Historical and current information from this episode of care organized as a timeline	√
**Diagnostic Assessment**	**8a**	Diagnostic testing (such as PE, laboratory testing, imaging, surveys).	√
	**8b**	Diagnostic challenges (such as access to testing, financial, or cultural)	√
	**8c**	Diagnosis (including other diagnoses considered)	√
	**8d**	Prognosis (such as staging in oncology) where applicable	√
**Therapeutic Intervention**	**9a**	Types of therapeutic intervention (such as pharmacologic, surgical, preventive, self-care)	√
	**9b**	Administration of therapeutic intervention (such as dosage, strength, duration)	√
	**9c**	Changes in therapeutic intervention (with rationale)	√
**Follow-up and Outcomes**	**10a**	Clinician and patient-assessed outcomes (if available)	√
	**10b**	Important follow-up diagnostic and other test results	√
	**10c**	Intervention adherence and tolerability (How was this assessed?)	√
	**10d**	Adverse and unanticipated events	√
**Discussion**	**11a**	A scientific discussion of the strengths AND limitations associated with this case report	√
	**11b**	Discussion of the relevant medical literature **with references**.	√
	**11c**	The scientific rationale for any conclusions (including assessment of possible causes)	√
	**11d**	The primary "take-away" lessons of this case report (without references) in a one paragraph conclusion	√
**Patient Perspective**	**12**	The patient should share their perspective in one to two paragraphs on the treatment(s) they received	√
**Informed Consent**	**13**	Did the patient give informed consent? Please provide if requested	**Yes** ☑ **No** ☐

Supplementary Figure 1. CARE check list

Taipei Medical University Hospital Department of Neurology
Electrodiagnostic Laboratory
Nerve Conduction & EMG / EP Report

Patient ID: Date of Exam:

Patient Name: Gender:
Height: Weight: Birthday:
Physician: Age:
Note:

Motor Nerve Conduction:

Nerve and Site	Lat. (ms)	Amp. (mV)	Lat.Diff. (ms)	Dist. (mm)	C.V. (m/s)
**Median.L**					
Wrist	3.0	8.6	3.0	80	
Elbow	6.7	7.7	3.7	190	**51**
**Ulnar.L**					
Wrist	2.3	7.4	2.3	80	
Above elbow	6.4	6.8	4.1	260	**63**
**Median.R**					
Wrist	3.4	7.5	3.4	80	
Elbow	7.0	5.5	3.6	200	**56**
**Ulnar.R**					
Wrist	2.7	7.1	2.7	80	
Above elbow	6.6	6.4	3.9	250	**64**

F-Wave Studies

Nerve	M-Latency ms	F-Latency ms
Median.L	3.4	**23.9**
Ulnar.L	2.6	**24.8**
Median.R	3.9	**24.3**
Ulnar.R	2.7	**25.7**

Sensory Nerve Conduction:

Nerve and Site	Onset Lat. (ms)	Peak Lat. (ms)	Amp. (μV)	Lat. Diff. (ms)	Dist. (mm)	C.V. (m/s)
**Median.L**						
Wrist	2.5	3.2	40	2.5	140	**56**
Elbow	5.9	6.6	24	3.4	190	**56**
**Ulnar.L**						
Wrist	2.4	3.0	24	2.4	140	**59**
Elbow	6.0	6.9	13	3.6	250	**69**
**Median.R**						
Wrist	2.8	3.5	37	2.8	140	**50**
Elbow	5.7	6.4	25	2.9	200	**69**
**Ulnar.R**						
Wrist	2.4	3.1	30	2.4	140	**58**
Elbow	5.9	6.8	20	3.5	240	**69**

Conclusions:
MNCV: within normal limit
SNCV: within normal limit
F-wave: within normal limit
The NCV study is essentially normal. Please correlate with clinical features.

(*Abbreviations: MNCV: motor nerve conduction velocity; NCV: nerve conduction velocity; SNCV: sensory nerve conduction velocity)

Supplementary Figure 2. Examination of motor nerve conduction and sensory nerve conduction. The nerve conduction velocity(NCV) study is essentially normal.
