# Supplementary figures and images for: Exploring the concept of surgical transition: surgical activity in the light of economic development in Sierra Leone, Liberia, Ghana and India
Source: Front Surg. 2025 Aug 15;12:1629828. doi: 10.3389/fsurg.2025.1629828 (PMC12394221; doi:10.3389/fsurg.2025.1629828)

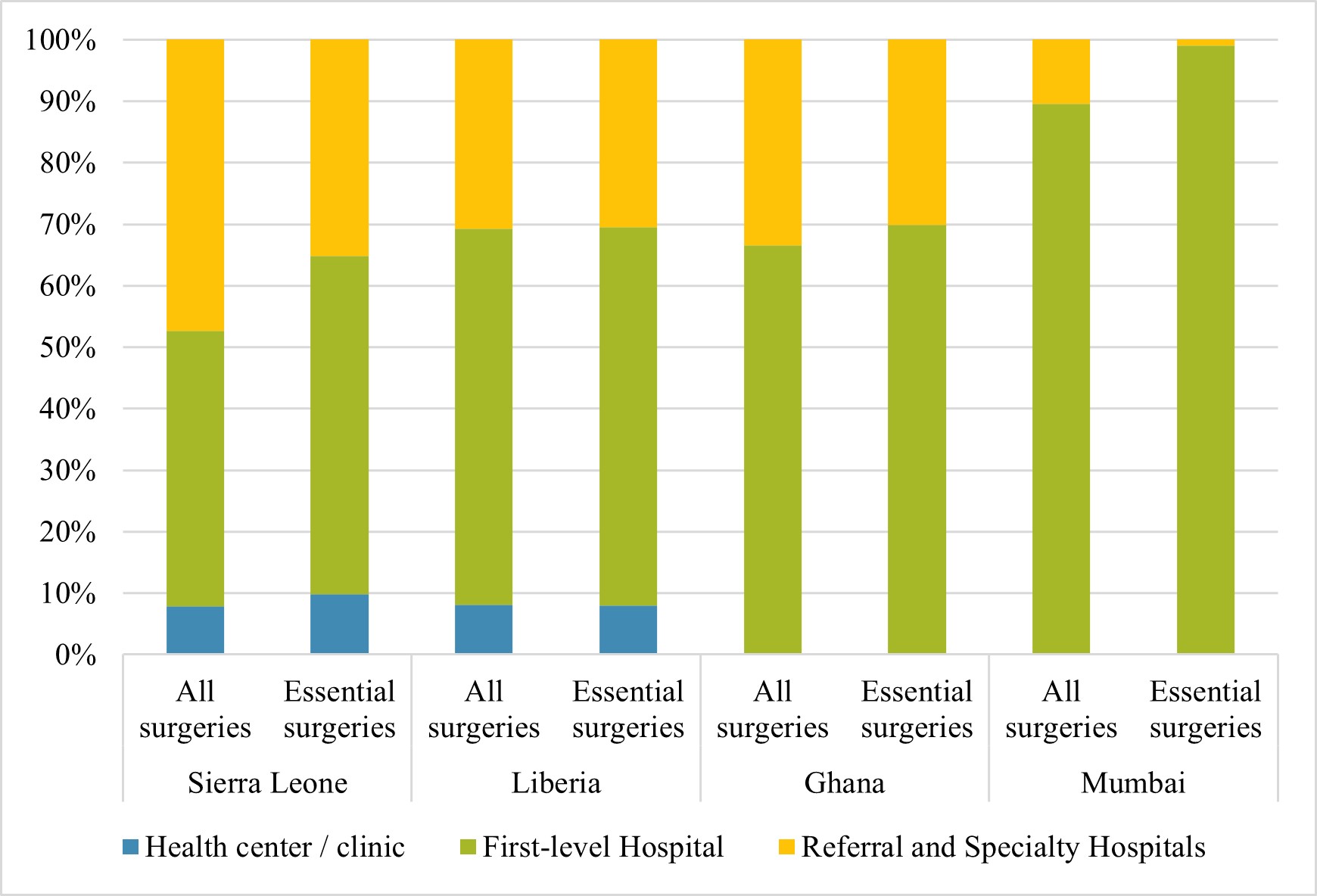

Supplement: Supplementary file 2 [file Image1.jpg]
